# Case report of avascular necrosis of the glans penis after PAE embolization

**DOI:** 10.1186/s12894-023-01201-0

**Published:** 2023-03-04

**Authors:** Jun Liu, Yang-Yang Gong, Can Ran, Hong-Jian Liu, Ding-Sheng Zeng, You-Gang Feng

**Affiliations:** Department of Urology, Suining Central Hospital, 127 Desheng West Road, Suining, 629000 Sichuan China

**Keywords:** Benign prostatic hyperplasia, Prostatic artery embolism, Penile ischemic necrosis, Gross hematuria, Case report

## Abstract

**Background:**

Prostate artery embolization (PAE) is a relatively safe and effective alternative method for the treatment of lower urinary tract symptoms secondary to benign prostatic hyperplasia. The adverse events caused by PAE are primarily mild, including urinary tract infection, acute urinary retention, dysuria, fever, etc. Severe complications, such as nontarget organ embolism syndrome or penile glans ischemic necrosis, are rare. Here, we report a case of severe ischemic necrosis of the glans penis after PAE and review the literature.

**Case presentation:**

An 86-year-old male patient was admitted to the hospital due to progressive dysuria with gross hematuria. The patient underwent placement of a three-way urinary catheter to facilitate continuous bladder flushing, hemostasis, and rehydration. After admission, his hemoglobin decreased to 89 g/L. After an examination, the diagnosis was benign prostatic hyperplasia with bleeding. During communication with the patient regarding treatment, he requested prostate artery embolization due to his advanced age and concomitant disease status. He underwent bilateral prostate artery embolization under local anesthesia. His urine gradually turned clear. However, on the 6th day after embolization, the glans gradually showed ischemic changes. On the 10th day, there was partial necrosis and blackening of the glans. The glans completely healed, and the patient was able to urinate smoothly on the 60th day after local cleaning and debridement, the administration of pain relief, anti-inflammatory and anti-infection agents, and external application of burn ointment.

**Conclusion:**

Penile glans ischemic necrosis after PAE is rare. The symptoms include pain, congestion, swelling, and cyanosis in the glans.

## Background

Prostatic artery embolization (PAE) is a safe and effective method to treat lower urinary tract symptoms secondary to benign prostatic hyperplasia (BPH). This procedure is especially suitable for those with a large prostate, elderly patients with intractable prostate bleeding, or BPH patients concerned with sexual function [1]. Minor adverse events caused by PAE include urinary tract infection, acute urinary retention, dysuria, and fever. Serious complications, such as nontarget organ embolism syndrome (including bladder ischemia), ischemic proctitis, and penile glans ischemia, are relatively rare [2]. Our department admitted a patient with benign prostatic hyperplasia with hemorrhage in July 2021. Severe avascular necrosis of the glans penis occurred after PAE. The patient recovered after active treatment.

## Case presentation

An 86-year-old male patient was admitted to the hospital due to progressive dysuria for three years and worsening hematuria for three days. The patient reported dysuria and hematuria with frequent urination and urgency but no obvious urination pain, low back pain, fever, nausea or vomiting, or use of anticoagulant drugs. The patient had previous hypertension, posterior circulation ischemia, carotid atherosclerosis with stenosis, peripheral neuropathy, and neuropathy in the lower limbs. Digital rectal examination revealed that the prostate was enlarged, the central groove was shallow, and the surface was smooth with no nodules. No tenderness was palpated, and no bloodstains were observed on the gloved finger. At hospital admission, the patient’s liver, kidney, and blood coagulation function were considered normal, apart from an HGB of 114 g/L. The tPSA level was 3.25 ng/ml. Routine urine testing demonstrated 1 + white blood cells. Before hospital admission, a Doppler ultrasound revealed blood clot in the bladder measuring 4.7 cm × 2.8 cm × 3.3 cm. The prostate was enlarged to approximately 86 g. There was 40 ml of residual urine in the bladder after urination. Computed tomography urography (CTU) scan indicated that the prostate volume was approximately 6.0 cm × 6.7 cm × 5.5 cm with several nodules and an area of high density inside. There were multiple diverticulum formations in the bladder. A mass with uneven enhancement was seen toward the right of the prostate (approximate size: 4.6 cm × 2.7 cm). Further prostatic MRI scans also revealed benign prostatic hyperplasia. The bone scan was negative. On day seven, a Doppler ultrasound indicated a bladder blood clot with a size of 6.6 cm × 4.9 cm × 6.7 cm.

After discussion, the cause of the patient’s bleeding was determined to be benign prostatic hyperplasia. After admission, the patient underwent placement of an indwelling three-way urinary catheter to facilitate continuous bladder irrigation, hemostatic drug to stop the bleeding, and piperacillin sodium and sulbactam sodium to fight infection. On the third day after admission, the patient’s urine color turned clear, and bladder irrigation was stopped. The catheter was removed on the fourth day after admission. The patient was able to urinate on his own without obvious gross hematuria. On day seven postadmission, the patient recurred with obvious gross hematuria accompanied by difficulty urinating and underwent placement of an indwelling three-was urinary catheter to facilitate continuous bladder flushing. The flushing fluid was dark red with a darker intermittent color. Hemostatic and fluid rehydration treatments were provided simultaneously.

**Fig. 1:**
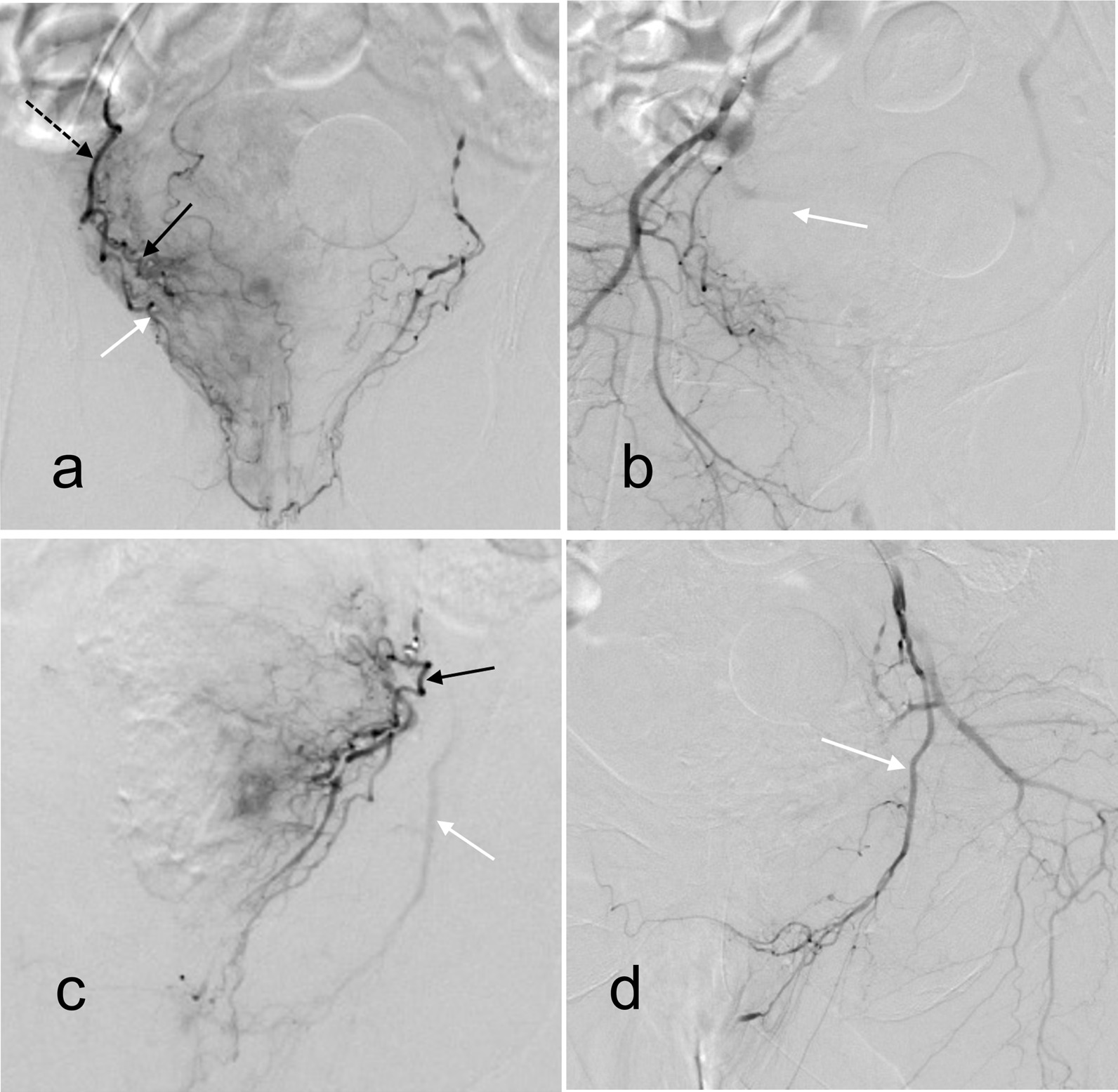
Imaging of bilateral prostatic artery embolism. **a** Both the right prostate artery (black solid arrow) and the dorsal penis artery (white arrow) originate from the internal pudendal artery (black dashed arrow). There are communicating branches between the right dorsal penis artery and the left dorsal penis artery. The left dorsal penis artery can be seen, suggesting that the right dorsal penis artery is the dominant blood supply. **b** After embolization of the right prostate artery, neither the prostate artery nor the dorsal penis artery were obstructed. **c** The left prostate artery (black solid arrow) and dorsal penis artery (white arrow) were separated. **d** After embolization, the left prostate artery was not developed, and the left dorsal penis artery was developed

As the patient is elderly and has underlying diseases, routine transurethral resection of the prostate carries a high risk. The patient agreed to prostate embolization. The interventional department performed bilateral prostatic artery embolization under local anesthesia (Fig. 1). After embolization, the patient’s urine color was clearer, but the urinary catheter was repeatedly blocked due to an old blood clot in the bladder. The urinary catheter to facilitate could not drain smoothly. The day after embolization, the bladder blood clot was cleared, and partial prostatectomy was performed through the urethra under spinal anesthesia in the operating room. After the operation, the patient’s urine color was clear, and gross hematuria did not recur.

On the second day after prostate embolization, the patient gradually developed glans pain, burning, and discomfort in the urethra and perineum. The glans was swollen with surface ecchymoses on day six postembolization. The patient was given a combination of oral and intravenous analgesics for pain relief and topical potassium permanganate and saline wet compresses, but the skin on the glans did not improve significantly.

**Fig. 2:**
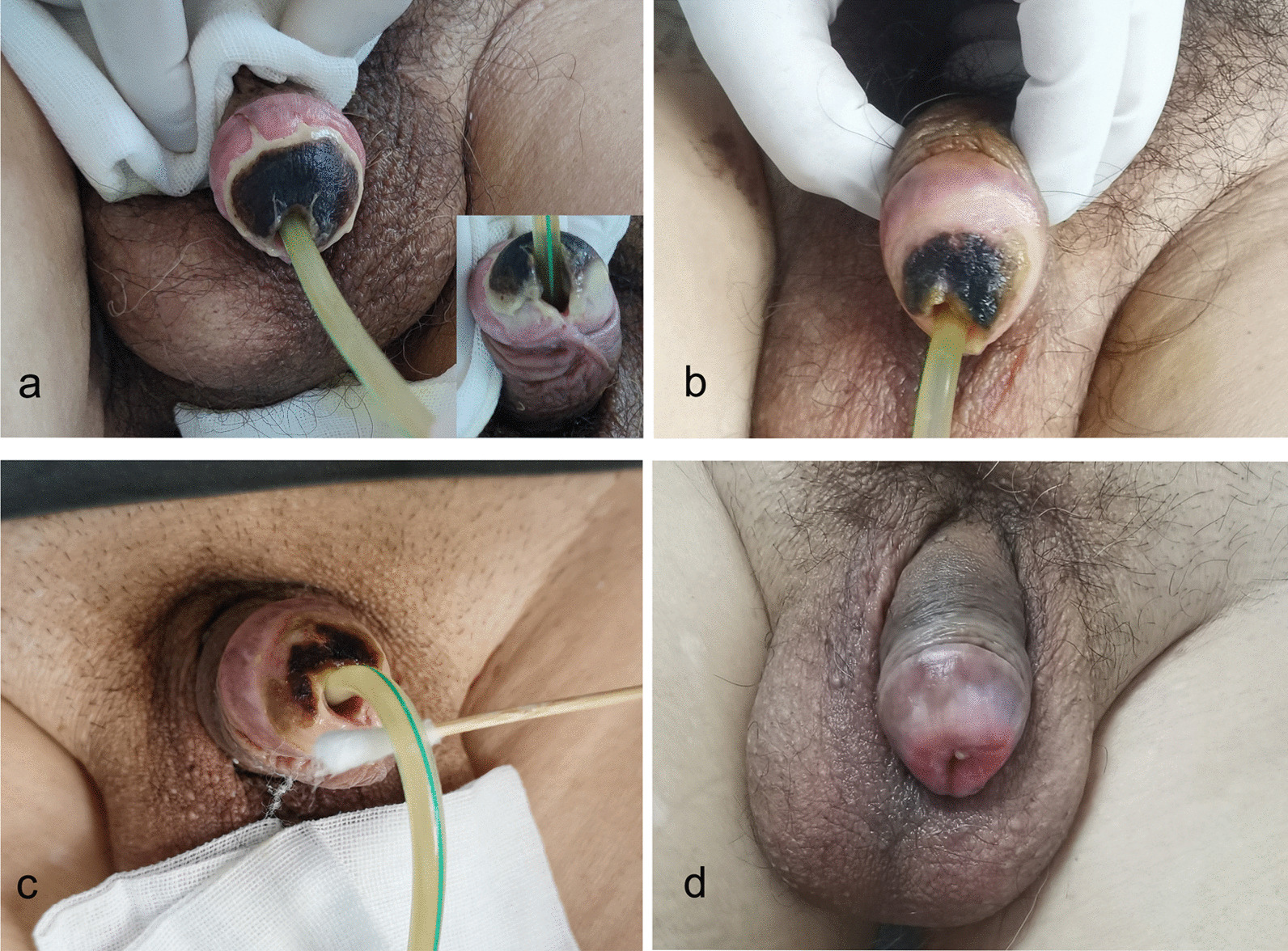
Evolution of avascular necrosis of the glans penis after prostatic artery embolization. **a** 10 days after the operation; **b** 30 days after the operation; **c** 40 days after the operation; and **d** 60 days after the operation

On the tenth day after embolization, most of the skin on the glans was crusted and black (Fig. 2a). A burn physician diagnosed avascular necrosis of the glans and recommended surgical removal of the scab. However, the patient refused. The glans was cleaned with saline and a new rehabilitation solution twice a day. The crust gradually softened and was removed with a blade. We first utilized lidocaine cream to relieve pain and then used burn cream to promote wound healing. Approximately 30 days after embolization, most of the crust on the glans had fallen off, and the base was visible. There was obvious granulation tissue, the wound was pink, and a small scab was noted around the urethral orifice. The mucous membrane around the urethral orifice was white (Fig. 2b, c). Approximately 60 days after embolization, the glans had completely healed (Fig. 2d), there was no obvious burning or discomfort in the urethra or scrotum, and urination was unobstructed. Three months after the operation, the prostate size, IPSS, residual urine and maximum urinary flow rate were 4.8 cm × 5 cm × 4.1 cm (approximately 51 g), 7 points, 20 ml and 16 ml/s, respectively. The patient was satisfied with the treatment outcome.

## Discussion and conclusions

Serious adverse events such as avascular necrosis of the penis after PAE are relatively rare. The nontargeted embolization can cause intravascular particles to flow to the adjacent penile artery, causing ischemic necrosis of the penis [3]. Most of the prostatic arteries are irregular, and they often originate from multiple blood supplies, including the inferior vesical artery, internal pudendal artery, and obturator artery. The prostatic artery has extensive anastomotic branches.

Improper prostatic artery embolization can lead to target organ embolism, triggering related adverse events. Therefore, correct identification of the prostatic artery and superselective cannulation embolization during angiography can reduce complications. In addition, a preoperative CTA examination of the prostate vessels can also assess the prostate’s vascular condition. A vascular catheter can be placed at the distal end of the vessel, or protective coil embolization can be performed if vascular anastomosis is found [4].

In this case, the right prostatic artery and dorsal penis artery were both derived from the internal pudendal artery, and the right dorsal penis artery was the dominant blood supply for the penis (Fig. 1a). Because the catheter did not enter the right prostatic artery, embolization occurred in the internal pudendal artery (Fig. 1b), resulting in false embolism of the dorsal penis artery and finally penile ischemia and necrosis. On the contrary, the catheter smoothly entered the left prostate artery for embolization(Fig. 1c), while the left dorsal penile artery developed well. (Fig. 1d).

Patients with avascular necrosis of the penis after PAE reportedly present with severe pain in the glans or penis, dysuria, and hematuria. Some patients will have erectile dysfunction. This patient gradually developed ischemic changes in the glans penis the second day after embolization and complained of severe local pain, accompanied by a burning sensation in the urethra and perineum. The patient required strong analgesics, cold compresses on the scrotum. The symptoms rebounded after a few hours. Local bruising and blackening of the glans gradually appeared on the sixth day after surgery. This bruising spread to the entire glans, accompanied by whitening of the mucous membrane around the urethral opening and local secretions.

The local pain symptoms were slightly relieved. However, daily oral and topical pain relief medications were required. Therefore, severe complications of penile avascular necrosis should be considered in patients with benign prostatic hyperplasia, severe pain in the penis or urethra, congestion and swelling of the glans, and gradual bruising and blackening after PAE.

At present, there is no standard treatment for avascular necrosis of the penis after PAE. Eric Chung reported that hyperbaric oxygen achieved good results in patients with penile ischemic necrosis. This treatment increases oxygen flow in local tissues, which can prevent inflammation, promote new blood vessel formation, strengthen fibroblasts and lymphocytes, and enhance macrophage activity, thereby promoting tissue and wound repair [5]. Cornelis used PAE to treat a 60-year-old BPH patient with penile ischemia changes 24 h after surgery. The patient immediately took tadalafil at 5 mg/day and acetylsalicylic acid at 100 mg/day. The patient’s condition normalized after seven days of treatment. Tadalafil relaxes the penile arteries and the smooth muscles of the penile cavernous body, increasing penile blood flow and promoting revascularization. Acetylsalicylic acid is used to prevent new thrombosis [6].

Nestor Kisilevzky used PAE to treat a 58-year-old BPH patient. The patient had ischemic changes on the dorsal side of the glans on day eight postoperation. Black necrosis on the dorsal side of the glans appeared on the 11th day. The patient was given oral ciprofloxacin at 1 g/day and fusidic acid cream and potassium permanganate twice a day. The glans completely healed on the 40th day after the operation [3]. In the initial stage of penile ischemic necrosis, the patient was provided anti-inflammatory and analgesic agents and local wet saline compresses.

When glans necrosis and black scabs appeared, the patient took Kangfuxin and alternated normal saline wet compresses every day to keep the area clean. Oral levofloxacin at 0.1 g/day was provided for anti-infection treatment. After the crust gradually softened, it was removed, and lidocaine cream and burn ointment were applied to the base twice a day to relieve pain and promote wound healing. By the 30th day after the operation, most of the scabs had fallen off, and fresh granulation tissue was visible. On the 60th day, the wound was completely healed, with smooth urination. A good effect was achieved.

PAE is a relatively safe and effective method to treat patients with high-risk, large-volume prostate hyperplasia and intractable bleeding of prostate. This procedure can be used as an alternative to conventional, minimally invasive surgical prostate treatment. However, nontargeted embolization can have serious consequences, such as avascular necrosis of the penis. Active treatment is required for this complication, which can often be cured by conservative treatment.

## Data Availability

Not applicable.

## Abbreviations

HGB: Hemoglobin
tPSA: Total prostate antigen
CTU: Computed tomography urography
MRI: Magnetic resonance imaging
CTA: Computed tomography angiography
